# Ultrasound-guided minimally invasive thread release of Guyon’s canal: initial experience in cadaveric specimens

**DOI:** 10.1186/s41747-024-00456-y

**Published:** 2024-05-08

**Authors:** Suren Jengojan, Philipp Sorgo, Gregor Kasprian, Johannes Streicher, Gerlinde Gruber, Veith Moser, Gerd Bodner

**Affiliations:** 1 Department of Biomedical Imaging and Image-Guided Therapy, Division of Neuroradiology and Musculoskeletal Radiology, Medical University of Vienna, Waehringer Gürtel 18-20, 1090 Vienna, Austria; 2 Department of Anatomy and Developmental Biology, Karl Landsteiner University of Health Sciences, Dr.-Karl-Dorrek-Straße 30, 3500 Krems, Austria; 3 Department of Trauma Surgery, Lorenz Boehler Hospital, Donaueschingenstraße 13, 1200 Vienna, Austria; 4 Neuromuscular Imaging Ordinationszentrum Döbling, Heiligenstaedter Straße 55-63, 1190 Vienna, Austria

**Keywords:** Decompression, Interventional, Ulnar nerve compression syndromes, Ultrasonography, Wrist

## Abstract

**Objective:**

Guyon’s canal syndrome is caused by compression of the ulnar nerve at the wrist, occasionally requiring decompression surgery. In recent times, minimally invasive approaches have gained popularity. The aim of this study was to assess the efficacy and safety of ultrasound-guided thread release for transecting the palmar ligament in Guyon’s canal without harming surrounding structures, in a cadaveric specimen model.

**Methods:**

After ethical approval, thirteen ultrasound-guided thread releases of Guyon’s canal were performed on the wrists of softly embalmed anatomic specimens. Cadavers showing injuries or prior operations at the hand were excluded. Subsequently, the specimens were dissected, and the outcome of the interventions and potential damage to adjacent anatomical structures as well as ultrasound visibility were evaluated with a score from one to three.

**Results:**

Out of 13 interventions, a complete transection was achieved in ten cases (76.9%), and a partial transection was documented in three cases (23.1%). Irrelevant lesions on the flexor tendons were observed in two cases (15.4%), and an arterial branch was damaged in one (7.7%). Ultrasound visibility varied among specimens, but essential structures were delineated in all cases.

**Conclusion:**

Ultrasound-guided thread release of Guyon’s canal has shown promising first results in anatomic specimens. However, further studies are required to ensure the safety of the procedure.

**Relevance statement:**

Our study showed that minimally invasive ultrasound-guided thread release of Guyon’s canal is a feasible approach in the anatomical model. The results may provide a basis for further research and refinement of this technique.

**Key points:**

• In Guyon’s canal syndrome, the ulnar nerve is compressed at the wrist, often requiring surgical release.

• We adapted and tested a minimally invasive ultrasound-guided thread release technique in anatomic specimens.

• The technique was effective; however, in one specimen, a small anatomic branch was damaged.

**Graphical Abstract:**

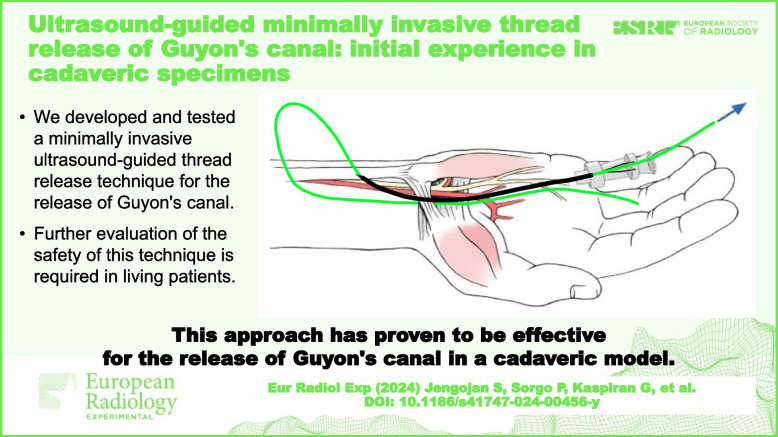

## Introduction

Guyon’s canal syndrome is a compression neuropathy of the ulnar nerve at the wrist where it passes through Guyon’s canal [1, 2]. This fibro-osseous tunnel is located on the ulnar side of the wrist and contains the ulnar artery and ulnar nerve. It is bordered by the flexor retinaculum, hypothenar musculature, and palmar carpal ligament. Clinical manifestations may include sensory or motor symptoms, such as weakness, tingling, or pain [3]. In addition to clinical tests, electroneurophysiological tests, magnetic resonance imaging (MRI), and high-resolution ultrasound of the wrist can aid in the diagnosis of this condition [4, 5]. The literature discusses various causes of Guyon’s canal syndrome, including ganglion cysts and idiopathic causes, which are among the most common etiologies [3]. However, tumors, repetitive trauma, aberrant muscles, and ulnar artery malformations may also cause this condition [6, 7]. Conservative therapies include instructing the patient to avoid stressing the wrist and splinting [4]. If these treatments are not sufficient, open surgical release of the canal or surgical treatment of the underlying condition is the therapy of choice [1, 4].

In recent years, a novel surgical approach for peripheral compression syndromes has been described in the carpal tunnel [5, 8, 9], cubital tunnel [10], trigger finger [11, 12], and compartment syndrome [13]: ultrasound-guided thread release (USGTR). Under ultrasound guidance, a cutting thread is looped around the targeted structure and used as a saw to release the compression [5]. High-resolution ultrasound is the method of choice for diagnosing compression neuropathies such as carpal tunnel syndrome and cubital tunnel syndrome. Similarly, Guyon’s canal can be visualized well by high-resolution ultrasound [14, 15]. Therefore, we postulate that USGTR is a feasible technique for the release of Guyon’s canal. The purpose of this anatomical study was to investigate whether the USGTR can be used for effective transection of the palmar ligament in the Guyon’s canal while avoiding damage to surrounding structures.

## Materials and methods

The study was submitted to and approved by the Commission for Scientific Integrity and Ethics of the Karl Landsteiner University of Health Sciences (vote number: 1052/2021).

Prior to the interventions, one cadaveric hand was excluded due to pre-mortem wrist surgery. Thirteen ultrasound-guided thread releases of Guyon’s canal were performed on softly embalmed anatomic specimens, using Thiel’s method of conservation [16]. This method of conservation is a sound model for ultrasound applications [17–19]. All interventions were performed by two neuromuscular radiologists with 25 (G.B.) and 8 (S.J.) years of experience in interventional musculoskeletal ultrasound. Cadavers were excluded prior to the intervention if they had confounding features, such as trauma or prior surgery at the wrist. The intervention was performed with 20-gauge, 10-cm spinal needles and a braided, medical quality stainless-steel cutting thread. To increase the visibility of the thread’s path after dissection, the threads were soaked for several hours with hematoxylin stain prior to the interventions. A GE Logiq E10s ultrasound system (GE Healthcare, Milwaukee) with broadband linear probes (6–22 MHz) was used. Subsequently, the specimens were dissected by anatomists J.S. and P.S. (30 years and 4 years of anatomical dissection experience, respectively). To assess each intervention, we developed a scoring system for different parameters. Ultrasound visibility, the outcome of the intervention, and possible damage to adjacent structures (nerves, tendons, vessels) were described using a three-point scale for each category (Table 1). Assessment of outcome and damage to adjacent structures was performed and discussed between all authors after the dissection, whereas ultrasound visibility was defined by both expert radiologists (G.B. and S.J.). All experiments were conducted between June and November of 2021 at the anatomical lab of the Karl Landsteiner University for Health Sciences in Austria. No complex statistical analysis was required for this study.

**Table 1:** Breakdown of the scoring system used to assess each intervention

Ultrasound visibility		
1	2	3
Rough identification of essential structures possible, no exact borders definable	Identification of essential structures possible, borders of most structures definable	Clear visibility of all essential structures
Outcome		
1	2	3
Less than three-quarters of the PCL transected	Three or more quarters of the PCL transected	Complete transection of the PCL
Safety		
1	2	3
Damage to macroscopic neurovascular structures	Unnecessary damage to surrounding connective tissue	No or minimal damage to adjacent structures

### Ultrasound-guided thread release protocol

**Fig. 1:**
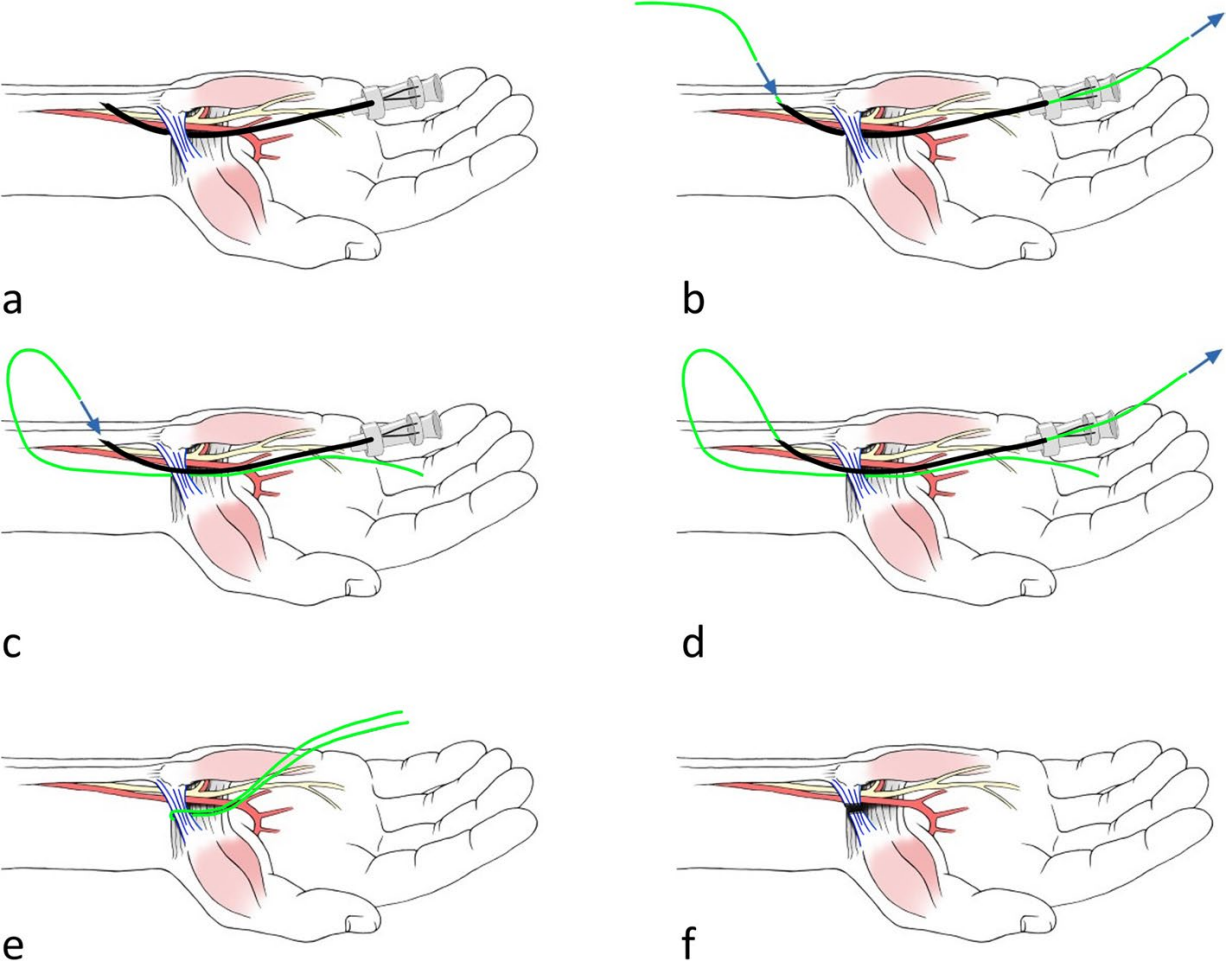
Minimally invasive ultrasound-guided release protocol; In the first step (**a**), a spinal needle is placed deep to the palmar carpal ligament (PCL), and the thread is inserted (**b**). Then, the needle is removed and reinserted superficial to the PCL, using the same incision hole (**c**). The thread is looped around the PCL (**d**), and the PCL is transected after removal of the needle (**e**, **f**)

We based our protocol on the work of Guo et al. [5] and adapted the release of the carpal tunnel for Guyon’s canal. Our approach is illustrated in Fig. 1. The carpal bones, palmar ligament, ulnar artery, and ulnar nerve with its branches were identified. Just before reaching the hook of the hamate, the ulnar nerve and its superficial and deep branches within Guyon’s canal were visualized. As the deep branch descends, the superficial branch continues distally within Guyon’s canal, running along the ulnar aspect of the palm and eventually branching off to the fifth digit.

**Fig. 2:**
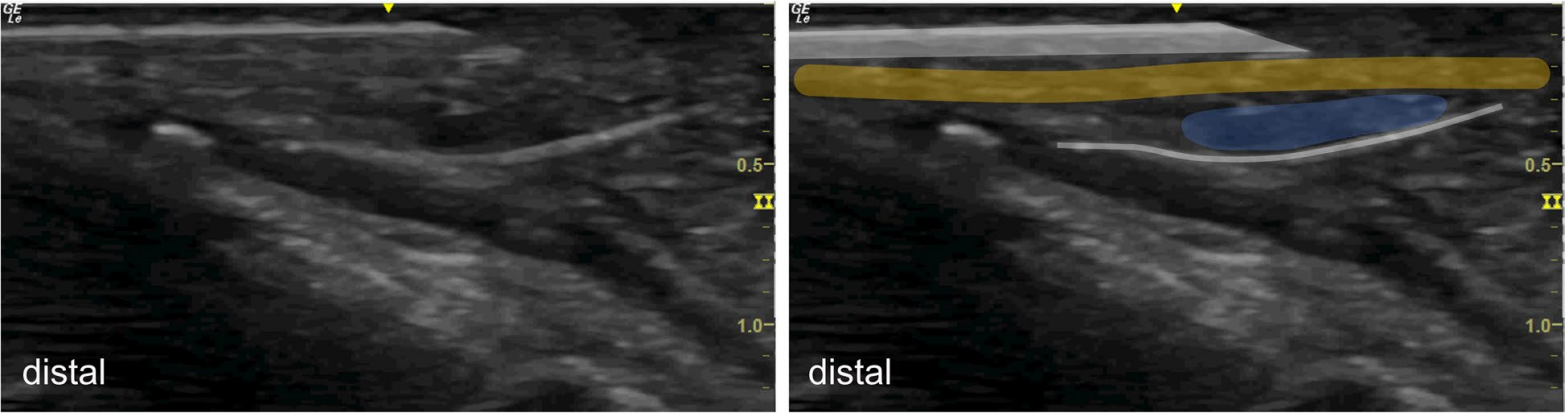
Placement of the thread loop using hydro dissection. The thread (white line) is already placed beneath the PCL (highlighted in orange). To place the thread above the PCL, the needle (highlighted with white contours) is placed above the PCL. Fluid accumulation due to hydro dissection is illustrated in blue

**Fig. 3:**
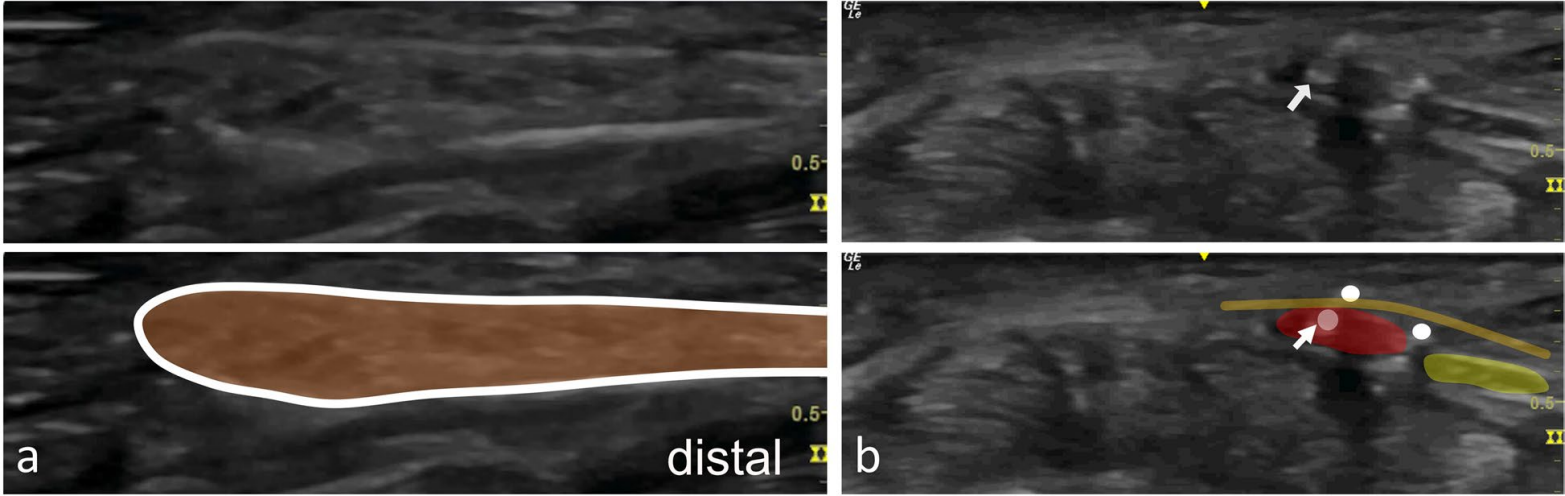
Final position of the thread in the long (**a**) and short (**b**) axis. Final position of the thread in the short axis in the left hand of a specimen. The thread (white line (**a**) and dots (**b**)) is placed in between the ulnar nerve (yellow) and the ulnar artery (red). The palmar carpal ligament is highlighted in orange (**a** and **b**). In this specimen, the ulnar artery shows extensive calcifications (white arrow)

After ultrasound visualization of the aforementioned structures, a spinal needle was inserted at the palm and placed deep to the palmar carpal ligament (PCL). Under continuous hydrodissection (injection of fluid to separate the PCL from the neurovascular structures within the canal), the needle was advanced towards the wrist exit and the thread was passed through the needle (Fig. 2). In the next step, the needle was withdrawn and repositioned superficial to the PCL. Under hydrodissection (this time aimed at separating the PCL from the superficial subcutaneous fat, small subcutaneous vessels, and nerve branches along the path), the needle was advanced towards the exit, utilizing the preexisting exit hole. The thread was inserted again, creating a thread loop around the PCL (Figs. 1d, e and 3). After ensuring the correct thread position, alternating forces were applied to the thread, using it as a saw to transect the PCL.

## Results

Out of 13 interventions, a complete transection was achieved in 10 cases (76.9%). Partial but almost complete transection (transection of more than 80% of the carpal ligament) was documented in three cases (23.1%).

Irrelevant lesions on the flexor tendons were observed in two cases (15.4%). However, a major complication occurred in one case (7.7%); a branch of the ulnar artery was truncated during the procedure, most likely due to its unusual position and poor visualization. Ultrasound visibility varied between specimens, but delineation of essential structures, a mandatory condition for the interventions, was possible in all cases. Severely reduced visibility was observed in 2 arms (15.4%). While complete transection of the carpal ligament without damage was achieved in one case (case 10), in the second case, an important structure, a branch of the ulnar artery, was damaged (case 13). Moderately reduced visibility was seen in 4 arms (cases 4–6 and 11), with irrelevant minor damage to the tendons occurring only in case 5. Incomplete transection occurred in cases 2, 9, and 12, with no reduction in ultrasound visibility in the last two cases (cases 9 and 12).

**Fig. 4:**
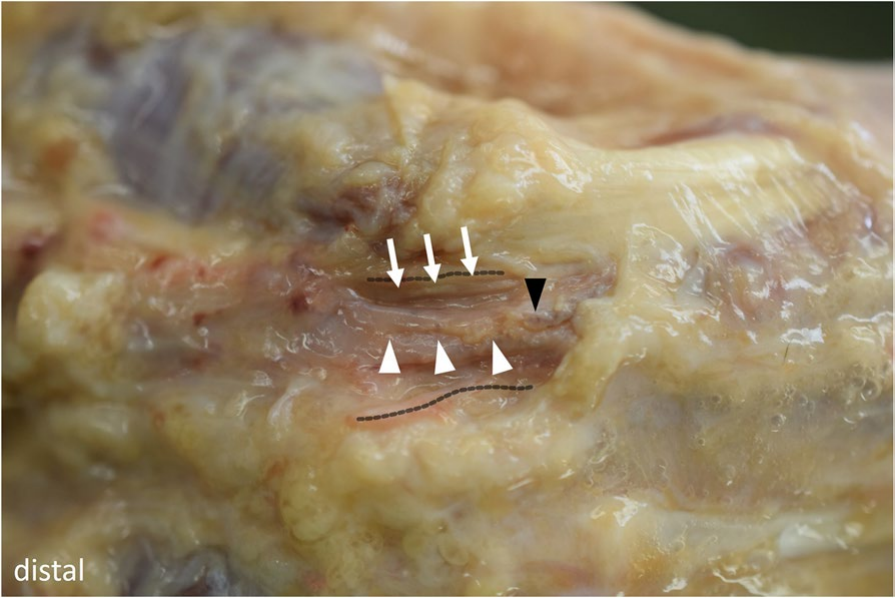
Dissection after a successful intervention + The ulnar artery (white arrowheads) and nerve (white arrows) are undamaged. The dashed lines represent the transected ligament stumps. Calcifications of the ulnar artery (black arrow) correspond with the ultrasound images in Fig. 3

Figure 4 shows an exemplary dissection after a successful intervention. Detailed results are illustrated in Table 2.

**Table 2:** List of interventions with their respective scores in all three categories

**Case number**	**1**	**2**	**3**	**4**	**5**	**6**	**7**	**8**	**9**	**10**	**11**	**12**	**13**
**US visibility**	3	3	3	2	2	2	3	3	3	1	2	3	1
**Outcome**	3	2	3	3	3	3	3	3	2	3	3	2	3
**Damage**	3	2	3	3	2	3	3	3	3	3	3	3	1

In cases number 2 and 5, the procedure resulted in damage to the ulnar carpal flexor tendon. In specimen 13, an arterial branch was dissected

## Discussion

We performed ultrasound-guided thread release of Guyon’s canal in 13 softly embalmed cadaveric specimens. Our results suggest that this relatively novel technique is effective and safe. To the knowledge of the authors, this is the first study on this topic in the scientific literature. Therefore, we cannot compare our results with preexisting studies on Guyon’s canal. However, previously published cadaveric studies on USGTR of the carpal tunnel [9, 20] yielded similar results to our study regarding outcome and damage to adjacent structures, except from the arterial damage in one case. In the cadaveric trials by Guo et al. [9] and Burnham et al. [20], USGTR of the carpal tunnel achieved a full transection of the transverse carpal ligament in 100% (11 out of 11) and 64.2% (9 out of 14). Both studies did not report damage to any vital structures.

Even though ultrasound visibility varied in our specimens, all interventions were technically possible. Severely reduced visibility was observed in two arms, with complete transection in one case and damage to a branch of the ulnar artery in the second case. Moderately reduced visibility was seen in four arms, with minor damage to the tendons occurring only in one case. Minor tendon injuries were observed in two cases. The first case was one of the first interventions, and in the second case, the ultrasound visibility was moderately reduced.

Incomplete transection occurred in three cases, with minor reduction in ultrasound visibility in one case. Although no clear reason can be given for the two incomplete transections during the intervention with good ultrasound visibility, it is possible that thin anatomical structures such as the carpal ligament in some embedded arms exhibit incomplete or reduced delineation in ultrasound, making precise transection more difficult. It seems reasonable and plausible that better ultrasound visibility might lead to better results. However, to statistically verify this, larger case numbers and comparative studies with fresh anatomical cadaver arms and patients with Guyon’s canal syndrome should be performed for further evaluation.

We want to emphasize that ultrasound visibility may also differ in patients, for example, in adipose or exsiccated patients, imaging can be suboptimal.

In one case (case 13), a palmar carpal branch of the ulnar artery was truncated by the cutting thread. It is a small branch originating from the ulnar artery, anastomosing with the palmar carpal branch of the radial artery, forming the palmar carpal arch [21]. In this case, the ultrasound visibility was severely reduced. Additionally, the unusual position of the branch (splitting from the ulnar artery at the level of Guyon’s canal) likely contributed to the complications of the intervention. We assume that irritations to vascular structures can be avoided in live patients, where more precise US examinations and tools such as doppler color US are available. We therefore suggest a thorough examination of the vascular situation prior to every procedure if clinical studies are performed using this technique.

As expected, damage to surrounding tissue and the skin was minimal. Similar advantages have already been described in endoscopic release of Guyon’s canal [22]. However, the potential risks of the endoscopic release, such as damage to the ulnar nerve and blood vessels, may also apply for USGTR. Therefore, we suggest that USGTR of the Guyon’s canal should only be performed by experienced physicians. Moreover, it is essential to definitively identify and visualize the ulnar nerve in the Guyon’s canal, including its two distal nerve branches, and to rule out any anatomical variations of vessels or nerves in the Guyon’s canal. Additionally, potential interactions between the palmar ulnar cutaneous and dorsal ulnar cutaneous nerves in the intervention area should be assessed to prevent nerve damage. Potential contraindications may be similar to endoscopic procedures at the canal, which are nerve compression by a solid tumor, severe hamate malunion, and pisiform dislocation [22]. Additionally, insufficient ultrasound visibility may also contraindicate the intervention.

Our study faces several limitations. Due to the limitations inherent in cadaver studies, we cannot conclusively demonstrate the feasibility of ultrasound-guided thread release (USGTR) of the Guyon canal in pathological patients. Abnormal structures within the canal, such as thickened ligaments, nerves, arteries, or adhesions, may impact the procedure’s outcomes. Therefore, we cannot ascertain whether USGTR of the Guyon’s canal effectively alleviates symptoms in living patients with Guyon’s canal syndrome. Despite the use of various minimally invasive methods such as needles, knives, retraction scalpels, or endoscopes in the past to release superficial ligaments or retinacula by others, we believe that this approach may be the most suitable for releasing Guyon’s canal. This is attributed to the narrow anatomical space filled with important neurovascular structures, necessitating the utilization of small-caliber instruments and precise visualization of the structures. Furthermore, hydrodissection enables direct and accurate mobilization of these critical structures [5, 13, 20, 22].

Even though Thiel embalmed cadavers are a suitable model for ultrasound-guided interventions, conditions may vary in patients. However, we believe that the average ultrasound visibility would be better *in vivo* and color doppler could be helpful for the detection of blood vessels. Furthermore, cadaver availability limits the sample size, decreasing the evidence level provided.

In conclusion, this study showed that high resolution ultrasound-guided thread release is a suitable technique for the release of Guyon’s canal in the anatomical model. However, due to the close spatial relationship between the palmar carpal ligament and the ulnar nerve and artery, a misplacement of the thread can have serious consequences for the patient. Therefore, we suggest that the technique needs to be refined, and more experience in cadaveric studies is required before clinical trials should be conducted.

## Data Availability

All data generated or analyzed during this study are included in this published article (and its supplementary information files).

## Abbreviations

PCL: Palmar carpal ligament
USGTR: Ultrasound-guided thread release
